# Transient Hypoperfusion to Ischemic/Anoxic Spreading Depolarization is Related to Autoregulatory Failure in the Rat Cerebral Cortex

**DOI:** 10.1007/s12028-021-01393-z

**Published:** 2021-12-02

**Authors:** Ákos Menyhárt, Dániel Péter Varga, Orsolya M. Tóth, Péter Makra, Ferenc Bari, Eszter Farkas

**Affiliations:** 1 Department of Medical Physics and Informatics, Faculty of Science and Informatics, Albert Szent-Györgyi Medical School, University of Szeged, Szeged, Hungary; 2 Cerebral Blood Flow and Metabolism Research Group, Hungarian Centre of Excellence for Molecular Medicine, University of Szeged, Szeged, Hungary; 3 Department of Cell Biology and Molecular Medicine, Faculty of Science and Informatics, Albert Szent-Györgyi Medical School, University of Szeged, Szeged, Hungary; 4 Institute for Stroke and Dementia Research, University Hospital, Ludwig Maximilians University Munich, Munich, Germany

**Keywords:** Cerebrovascular autoregulation, Cerebral blood flow, Ischemic stroke, Neurovascular coupling, Spreading depolarization, Spreading ischemia

## Abstract

**Background:**

In ischemic stroke, cerebral autoregulation and neurovascular coupling may become impaired. The cerebral blood flow (CBF) response to spreading depolarization (SD) is governed by neurovascular coupling. SDs recur in the ischemic penumbra and reduce neuronal viability by the insufficiency of the CBF response. Autoregulatory failure and SD may coexist in acute brain injury. Here, we set out to explore the interplay between the impairment of cerebrovascular autoregulation, SD occurrence, and the evolution of the SD-coupled CBF response.

**Methods:**

Incomplete global forebrain ischemia was created by bilateral common carotid artery occlusion in isoflurane-anesthetized rats, which induced ischemic SD (iSD). A subsequent SD was initiated 20–40 min later by transient anoxia SD (aSD), achieved by the withdrawal of oxygen from the anesthetic gas mixture for 4–5 min. SD occurrence was confirmed by the recording of direct current potential together with extracellular K^+^ concentration by intracortical microelectrodes. Changes in local CBF were acquired with laser Doppler flowmetry. Mean arterial blood pressure (MABP) was continuously measured via a catheter inserted into the left femoral artery. CBF and MABP were used to calculate an index of cerebrovascular autoregulation (rCBFx). In a representative imaging experiment, variation in transmembrane potential was visualized with a voltage-sensitive dye in the exposed parietal cortex, and CBF maps were generated with laser speckle contrast analysis.

**Results:**

Ischemia induction and anoxia onset gave rise to iSD and aSD, respectively, albeit aSD occurred at a longer latency, and was superimposed on a gradual elevation of K^+^ concentration. iSD and aSD were accompanied by a transient drop of CBF (down to 11.9 ± 2.9 and 7.4 ± 3.6%, iSD and aSD), but distinctive features set the hypoperfusion transients apart. During iSD, rCBFx indicated intact autoregulation (rCBFx < 0.3). In contrast, aSD was superimposed on autoregulatory failure (rCBFx > 0.3) because CBF followed the decreasing MABP. CBF dropped 15–20 s after iSD, but the onset of hypoperfusion preceded aSD by almost 3 min. Taken together, the CBF response to iSD displayed typical features of spreading ischemia, whereas the transient CBF reduction with aSD appeared to be a passive decrease of CBF following the anoxia-related hypotension, leading to aSD.

**Conclusions:**

We propose that the dysfunction of cerebrovascular autoregulation that occurs simultaneously with hypotension transients poses a substantial risk of SD occurrence and is not a consequence of SD. Under such circumstances, the evolving SD is not accompanied by any recognizable CBF response, which indicates a severely damaged neurovascular coupling.

## Introduction

Optimal neuronal function is critically dependent on blood supply matching metabolic requirements. Cerebral autoregulation ensures that the blood supply to the brain is constant despite changes in perfusion pressure, whereas neurovascular coupling adapts local cerebral blood flow (CBF) to metabolic needs generated by neuronal activity. In cerebral ischemic stroke, cerebral autoregulation and neurovascular coupling may become impaired, effecting the dysregulation of CBF, thereby aggravating the ongoing perfusion deficit instigated by the initial vascular obstruction. Ultimately, the weakening of cerebral autoregulation or neurovascular coupling exacerbate neuronal injury and impede tissue viability.

The loss of cerebrovascular autoregulation has been proposed as a relevant mechanism of secondary injury progression and worse functional outcome after ischemic stroke [1]. In particular, autoregulatory failure in malignant focal cerebral ischemia was found to be prevalent over the entire ischemic region, being most prominent in viable penumbra tissue [2]. In addition, the loss of cerebral autoregulation in large middle cerebral artery territorial stroke was coincident with malignant edema formation and predicted poor neurological outcome, in contrast with the benign course of the disease associated with preserved autoregulation [3]. Along with impaired cerebrovascular autoregulation, the efficacy of neurovascular coupling to passive sensory motor stimulation was also reported to decline with the increasing severity of ischemic stroke and correlated with poor clinical outcome [4]. The restricted CBF response to somatosensory stimulation may be linked to suppressed neuronal activity under ischemia, but the reduction of the CBF response appears to be disproportionally greater than the attenuation of neuronal activity as the severity of ischemia deepens [5].

According to the presently held view, the CBF response to spreading depolarization (SD) is governed by neurovascular coupling [6]. SD is an abrupt, transient loss of transmembrane ion gradients in a critical mass of cortical tissue, which propagates over gray matter at a rate of millimeters per minute, together with a multiphasic CBF response [6, 7]. In focal cerebral ischemia, in which SD has been recognized as a pathophysiological phenomenon, SD events are triggered repeatedly by metabolic supply–demand mismatch transients at the peri-infarct penumbra [8]. Subsequently, SDs traverse across the penumbra and are thought to recruit viable penumbra tissue to the infarcted core region [9–11]. The insufficiency of the associated CBF response may be critical in the harmful impact of SD. Notably, the insufficiency of the SD-coupled CBF response is more prevalent in aging as opposed to young experimental animals [12], which may be a contributing factor to the accelerated progression of infarct maturation in older patients with stroke [13].

Ideally, the CBF response to SD is composed of at least three subsequent elements of varying amplitude and duration. The CBF response starts with a brief hypoperfusion, which is promptly followed by a prominent, transient hyperemia. The CBF response is then concluded by a long-lasting oligemia [6]. The early hypoperfusion appears to be the phase most relevant for ischemic pathophysiology. While in optimally perfused brain tissue, the hyperemic element rules the CBF response, under ischemia, the early hypoperfusion phase gains ground on the subsequent hyperemia and may completely override the hyperemic element [14, 15]. The latter scenario is known as spreading ischemia, which develops as a result of inverse neurovascular coupling [16–18]. The SD-related hypoperfusion is most likely mediated by the high, vasoconstrictive concentration of extracellular K^+^ ([K^+^]_e_), especially at limited nitric oxide availability [19, 20]. As spreading ischemia propagates with SD over the penumbra zone, it enhances the metabolic crisis and jeopardizes the survival of the nervous tissue.

Intriguingly, spreading ischemia coupled with SD was found to be coincident with the loss of cerebral autoregulation in patients with traumatic brain injury and a case of subarachnoid hemorrhage [21, 22]. Intuitively, spreading ischemia may be concurrent with autoregulatory failure in ischemic stroke as well, but this has not been demonstrated yet. In addition, the causal relationship between autoregulatory dysfunction and SD or spreading ischemia has remained speculative, and experimental models are necessary to explore the interplay.

In the present study, we created penumbra-like ischemia in the rat cerebral cortex and investigated spontaneous SD evolution in response to supply–demand mismatch. We paid particular attention to the CBF response to SD and its relation to cerebrovascular autoregulation. Old rats were used in the study because spreading ischemia in the ischemic cerebral cortex is more likely to occur in old rats than in young rats [12]. In addition, ischemic stroke incidence is highest in the older patient population, therefore experimenting with old animals may yield results that have increased translational relevance. We report that the CBF drop with SD concomitant with autoregulatory dysfunction is passive flow reduction, following transient hypotension in our model, rather than spreading ischemia caused by active vasoconstriction.

## Methods

All applicable institutional and/or national guidelines for the care and use of animals were followed. The experimental procedures were approved by the National Food Chain Safety and Animal Health Directorate of Csongrád County, Hungary. The procedures were performed according to the guidelines of the Scientific Committee of Animal Experimentation of the Hungarian Academy of Sciences (updated Law and Regulations on Animal Protection: 40/2013. [II. 14.] Gov. of Hungary), following the European Union Directive 2010/63/EU on the protection of animals used for scientific purposes, and reported in compliance with the ARRIVE guidelines.

### Surgical Procedures

Old, adult, male Sprague–Dawley rats (Charles River Laboratories, 18 months old, 645 ± 100 g, *n* = 12) were used in this study. Standard rodent chow and tap water were supplied ad libitum. The animals were housed under constant temperature, humidity, and lighting conditions (23 °C, 12:12 h light/dark cycle, lights on at 7 a.m.). Animals were anesthetized with 1.5–2% isoflurane evaporated with N_2_O:O_2_ (70% and 30%, respectively) and allowed to breathe spontaneously through a head mask throughout the experiment. Body (core) temperature was kept at 37 °C with a feedback-controlled heating pad. Atropine (0.1%, 0.1 ml) was administered intramuscularly shortly before surgical procedures to avoid the production of airway mucus. Mean arterial blood pressure (MABP) was monitored continuously and arterial blood gases were checked regularly (i.e., during baseline, under ischemia, and under anoxia) via a catheter inserted into the left femoral artery. Level of anesthesia was controlled with the aid of MABP displayed live as experiments were in progress.

A midline incision was made on the neck and both common carotid arteries were gently separated from the surrounding tissue and the vagal nerves. Lidocaine (1%) was applied topically before opening the tissue layers during preparation. A silicone coated fishing line used as occluder was looped around the common carotid arteries for the later induction of cerebral ischemia.

For electrophysiological data acquisition (*n* = 11), two craniotomies (4–5 mm apart) were prepared in the right parietal bone by using a dental drill. The dura in each craniotomy was carefully removed, and the exposed brain surface was regularly rinsed with artificial cerebrospinal fluid (mM concentrations: 126.6 NaCl, 3 KCl, 1.5 CaCl_2_, 1.2 MgCl_2_, 24.5 NaHCO_3_, 6.7 urea, and 3.7 glucose bubbled with 95% O_2_ and 5% CO_2_ to achieve a constant pH of 7.4). For imaging (*n* = 1), the rostral cranial window was enlarged (4.5 × 4.5 mm) and closed by a microscopic cover glass to serve in vivo optical imaging, as described earlier [23]. The caudal craniotomy was created to have the opportunity to trigger SD with the topical application of 1 M KCl, should no spontaneous depolarization occur [24]. Retrospectively, this proved to be an unnecessary precaution, as all SDs analyzed in this study evolved spontaneously.

### Experimental Protocol

Isoflurane anesthesia for data acquisition was adjusted to and kept at 1.0–1.2%. Incomplete global forebrain ischemia was induced by the permanent occlusion of both common carotid arteries (two-vessel occlusion [2VO]), which readily gave rise to a single spontaneous SD (ischemic SD [iSD]). Oxygen was withdrawn from the anesthetic gas mixture 20–40 min later, creating a brief episode of anoxia, which produced anoxic/ischemic depolarization (aSD). The model has been intended to recapitulate the conversion of the ischemic penumbra to the irreversibly injured core region by the passage of the second SD (i.e., the aSD). The second event (the aSD) was triggered to worsen the metabolic challenge and promote the transformation of penumbra-like tissue to a severely injured state [25]. The condition of anoxia was resolved within 5 min by reoxygenation to avoid cardiac arrest, and then data acquisition was continued for 25 min.

### Local Field Potential, Extracellular Potassium, and CBF Measurements

Local field potential (LFP), [K^+^]_e_, and local changes to the CBF were acquired from the rostral craniotomy. Potassium sensitive microelectrodes were prepared, as reported earlier [20]. In brief, glass capillary microelectrode tips (outer diameter 10–12 μm) were filled with a liquid K^+^-ion exchanger membrane (Potassium ionophore I, cocktail A; Sigma), and the microelectrode shank was backfilled with 100 mM of KCl solution. Microelectrodes were calibrated in standard solutions of known K^+^ concentrations (1, 3, 5, 10, 30, 50, and 100 mM). K^+^-sensitive microelectrodes were lowered into the cortex together with microelectrodes (tip diameter = 20 μm) filled with 150 mM of NaCl and 1 mM of HEPES (4-(2-hydroxyethyl)-1-piperazineethanesulfonic acid). The signal of these microelectrodes was filtered in direct current (DC) potential mode (< 1 Hz) and served as the reference LFP signal for the K^+^-sensitive microelectrodes. An Ag/AgCl electrode placed under the skin of the animal’s neck was used as common ground. Microelectrodes were connected to custom made dual-channel electrometers (including AD549LH; Analog Devices, Norwood, MA) via Ag/AgCl leads. The reference electrode signal (i.e., the LFP) was subtracted from that of the K^+^-sensitive microelectrode by dedicated differential amplifiers and associated filter modules (NL106 and NL125; NeuroLog System; Digitimer Ltd, United Kingdom), which yielded potential variations related to changes in [K^+^]_e_. The recorded analog signals were converted and displayed live using a dedicated analog-to-digital converter (MP 150, Biopac Systems, Inc) at a sampling frequency of 1 kHz. Changes of [K^+^]_e_ were expressed in mV to be translated into mM concentration offline, using least squares linear regression [20].

CBF changes were monitored with a laser Doppler needle probe (Probe 403 connected to PeriFlux 5000; Perimed AB, Sweden) positioned next to the penetration site of the K^+^-sensitive microelectrode. The flow signal was digitized and displayed together with the DC potential and K^+^ signals, as described above (MP 150 and AcqKnowledge 4.2.0; Biopac Systems Inc, CA). The completed preparations were enclosed in a Faraday cage.

### Live, Optical Imaging of Changes in Cortical Transmembrane Potential and CBF

A voltage-sensitive dye (VS-dye, RH-1838; Optical Imaging Ltd, Rehovot, Israel) was dissolved in artificial cerebrospinal fluid and circulated in the closed cranial window to saturate the cortical tissue, as reported earlier [23]. For live imaging, the cortex was illuminated in stroboscopic mode (100 ms/s) with a high-power light emitting diode (625 nm peak wavelength; SLS-0307-A; Mightex, Pleasanton, CA, equipped with an emission filter 620–640 nm bandpass; 3RD620-640; Omega Optical Inc, Brattleboro, VT) and with a laser diode (HL6545MG; Thorlabs Inc, NJ; 120 mW; 660 nm emission wavelength) driven by a power supply (LDTC0520; Wavelength Electronics Inc, Bozeman, MT) set to deliver a 160-mA current [26]. Two identical CCD (Charged Coupled Device) cameras (resolution 1024 × 1024 pixels, Pantera 1M30; DALSA, Gröbenzell, Germany) were attached to a stereomicroscope (MZ12.5; Leica Microsystems, Wetzlar, Germany) equipped with a 1:1 binocular/video-tube beam splitter. The VS-dye fluorescence was captured with a camera equipped with a band pass filter (3RD 670–740; Omega Optical Inc). To create CBF maps by laser speckle contrast analysis (LASCA), raw speckle images were captured by the second CCD camera (1 frame/second; 2 ms for illumination and 100 ms for exposure). A dedicated program written in LabVIEW environment synchronized the illumination and camera exposures. CBF maps were generated from the corresponding raw images, as reported earlier [24]. Changes in VS-dye fluorescence intensity and CBF with time were extracted by placing regions of interest (ROIs) of 19 × 19-pixel size (~ 70 × 70 μm) at selected sites on the surface of cerebral cortex.

### Data Processing and Statistical Analysis

All variables (i.e., LFP, [K^+^]_e_, laser doppler flow signal, and MABP) were simultaneously acquired, displayed live, and stored using a personal computer equipped with a dedicated software (AcqKnowledge 4.2 for MP 150; Biopac Systems Inc, CA). Data analysis was conducted offline and was assisted by the inbuilt tools of AcqKnowledge 4.2 software. Three rats were excluded from analysis because of artifacts on the [K^+^]_e_ or the DC potential trace.

**Fig. 1:**
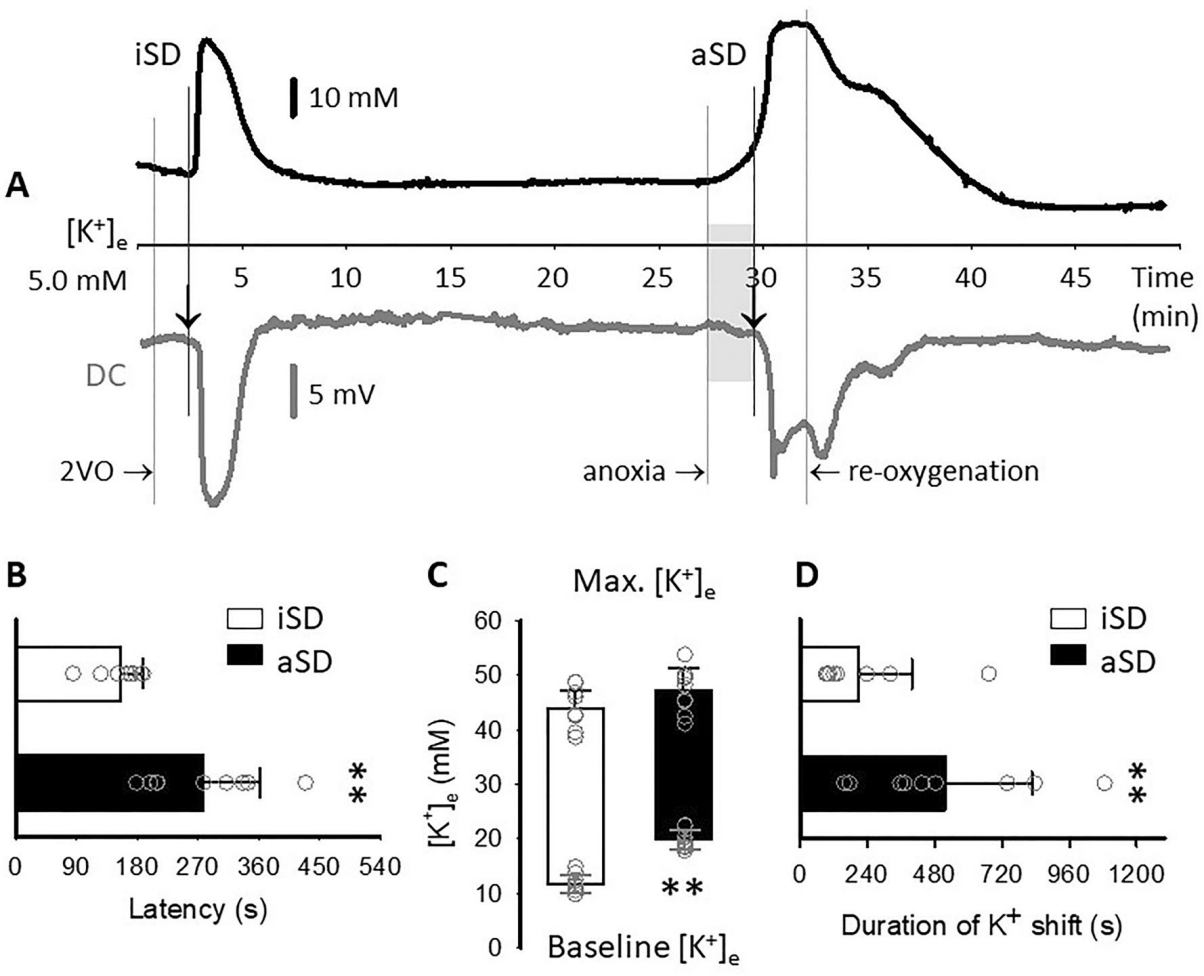
The distinct electrophysiological features of spontaneous spreading depolarization in response to ischemia induction (ischemic SD [iSD]) and anoxia (anoxic SD [aSD]). **a** Representative traces demonstrate the typical shift of extracellular potassium concentration ([K^+^]_e_) and the negative DC potential deflection with iSD and aSD. Arrows indicate event onset. In contrast with iSD, aSD was preceded by a slow, gradual deflection of [K^+^]_e_ and a parallel, slow negative shift of the DC potential trace (gray shading). **b** aSD occurred at a considerably longer latency with respect to anoxia onset than iSD with respect to ischemia induction (2VO). **c** Extracellular potassium concentration (base of bars) was significantly higher prior to aSD than iSD because of the slow [K^+^]_e_ elevation preceding aSD, shown with gray shading in Panel A. The absolute peak [K^+^]_e_ (top of bars) was also higher for aSD with respect to iSD. **d** The duration of the [K^+^]_e_ shift with the events was markedly longer with aSD compared with iSD. Data are given as mean ± stdev. Statistical analysis relied on a one-way analysis of variance model run in SPSS; *p* < 0.05* and *p* < 0.01**. 2VO, two-vessel occlusion, DC, direct current, [K^+^]_e_,  extracellular K^+^ concentration, stdev, standard deviation

To characterize iSD and aSD, several variables were measured in the DC potential and the synchronous [K^+^]_e_ traces. Note that the DC potential signature of SD can be used as a surrogate marker of [K^+^]_e_ changes, and the two traces were taken into consideration (as confirmation) for variables expressed in time (e.g., event latency) or to determine aSD onset (Fig. 1a). First, the latency of the SD events with respect to 2VO or anoxia initiation was determined. The onset of SD superimposed on a negative DC potential drift was taken at a sharp (> 1 mV/s) negative shift that occurred within minutes after ischemia or anoxia induction (Fig. 1a). Next, the peak amplitude of the events, the duration at half amplitude, the rates of depolarization, and the concomitant rise of [K^+^]_e_ were taken, as previously reported [23]. The rate of the slow DC potential (mV/s) and [K^+^]_e_ (mM/s) drift prior to aSD was calculated from the deflection from the level baseline to the onset of aSD. Finally, the amplitude of the hypoperfusion transients with iSD and aSD, as well as the duration of the hypoperfusion transients at half amplitude, were measured.

The status of cerebral autoregulation during the CBF responses to iSD and aSD were determined by calculating the cerebrovascular autoregulatory index (rCBFx) in MATLAB with Signal Processing and Image Processing Toolboxes (R2018b; MathWorks, Natwick, MA), as described in detail elsewhere [21]. Briefly, CBF and MABP were downsampled to 1 Hz. Then, consecutive 8-s averages were calculated with a sixth order median filter for each to eliminate high-frequency noise. Afterward, a Pearson correlation coefficient was calculated for every 40 data points yielding rCBFx in a temporal resolution of 320 s. Then, discrete data were transformed to continuous variable using linear regression. Section averages (e.g., baseline, before, and during SD) were measured on this time series of 1 Hz resolution. Intact cerebrovascular autoregulation was indicated by rCBFx < 0.3 [27]. Local CBF variations obtained by LDF or LASCA were expressed relative to baseline by using the average CBF value of the first 5 min of baseline (100%) and the recorded biological zero obtained after terminating each experiment (0%).

Quantitative data are given as mean ± standard deviation. Statistical analysis was conducted with the software SPSS (IBM SPSS Statistics for Windows, Version 22.0; IBM Corp, Armonk, NY). Data sets were evaluated by a one-way analysis of variance, followed by a Bonferroni post hoc test when appropriate. Level of significance was set at *p* < 0.05* or *p* < 0.01**. Appropriate statistical methods are provided in each Figure legend in detail.

## Results

### *Characterization of the *In Vivo* Model*

Successful ischemia induction was confirmed by a sudden drop of CBF to 22.2 ± 11.4% of baseline shortly after 2VO. CBF then stabilized around 37.8 ± 19.8% prior to anoxia initiation and was sustained at 33.5 ± 21.3% following the insult. Partial pressure of arterial blood O_2_ (pO_2_) varied in the normal range during ischemia (117.5 ± 31.7 vs. 107.7 ± 23.1 mm Hg, ischemia vs. baseline), and profoundly decreased to 34.4 ± 10.5 mm Hg under the episode of anoxia. The partial pressure of arterial CO_2_ (pCO_2_) varied within the physiological range throughout the experimental protocol (35.4 ± 12.9 vs. 41.3 ± 4.1 vs. 37.37.8 ± 4.0 mm Hg, anoxia vs. ischemia vs. baseline; *f* = 1.633, *p* = 0.211). MABP varied around 86 ± 19 mm Hg during baseline and displayed a gradual elevation over the ischemic period to 99 ± 18 mm Hg. The subsequent anoxia was promptly followed by a considerable reduction of MABP to 64 ± 24 mm Hg, whereas reoxygenation produced a transient MABP increase more than baseline, peaking at 105 ± 24 mm Hg. MABP then settled around 96 ± 22 mm Hg.

### Evolution of Spreading Depolarization Events, and Related Changes in CBF

Spreading depolarization events occurred spontaneously in response to ischemia and anoxia initiation (Fig. 1a). The latency with respect to the onset of the triggering insult proved to be longer for aSD than for iSD (277 ± 84 vs. 155 ± 33 s, respectively) (Fig. 1b). The onset of iSD was indicated by a sharp rise of [K^+^]_e_ and a concomitant sudden drop of the DC potential. In contrast, aSD was preceded by and started off from a slow, gradual elevation of [K^+^]_e_, (relative amplitude 11.8 ± 2.09 mM, duration 166 ± 46 s), accompanied by a matching negative drift of the DC potential (relative amplitude 2.5 ± 1.3 mV) (Fig. 1a, gray shading). Because of the slow elevation of [K^+^]_e_, aSD took off from a considerably higher [K^+^]_e_ than iSD (19.8 ± 1.7 vs. 11.7 ± 1.7 mM, respectively), whereas the absolute peak amplitude of the [K^+^]_e_ and DC potential shift with aSD and iSD were similar ([K^+^]_e_ 47.3 ± 4.1 and 44.1 ± 3.7 mM, respectively) (Fig. 1c). The rate of depolarization was significantly slower with aSD than with iSD ([K^+^]_e_ 0.62 ± 0.38 vs. 2.08 ± 0.53 mM/s, respectively; DC potential: − 0.55 ± 0.47 vs. − 1.17 ± 0.74 mV/s, respectively). Finally, aSD lasted characteristically longer than iSD (516 ± 311 vs. 208 ± 191 s, respectively) (Fig. 1d).

**Fig. 2:**
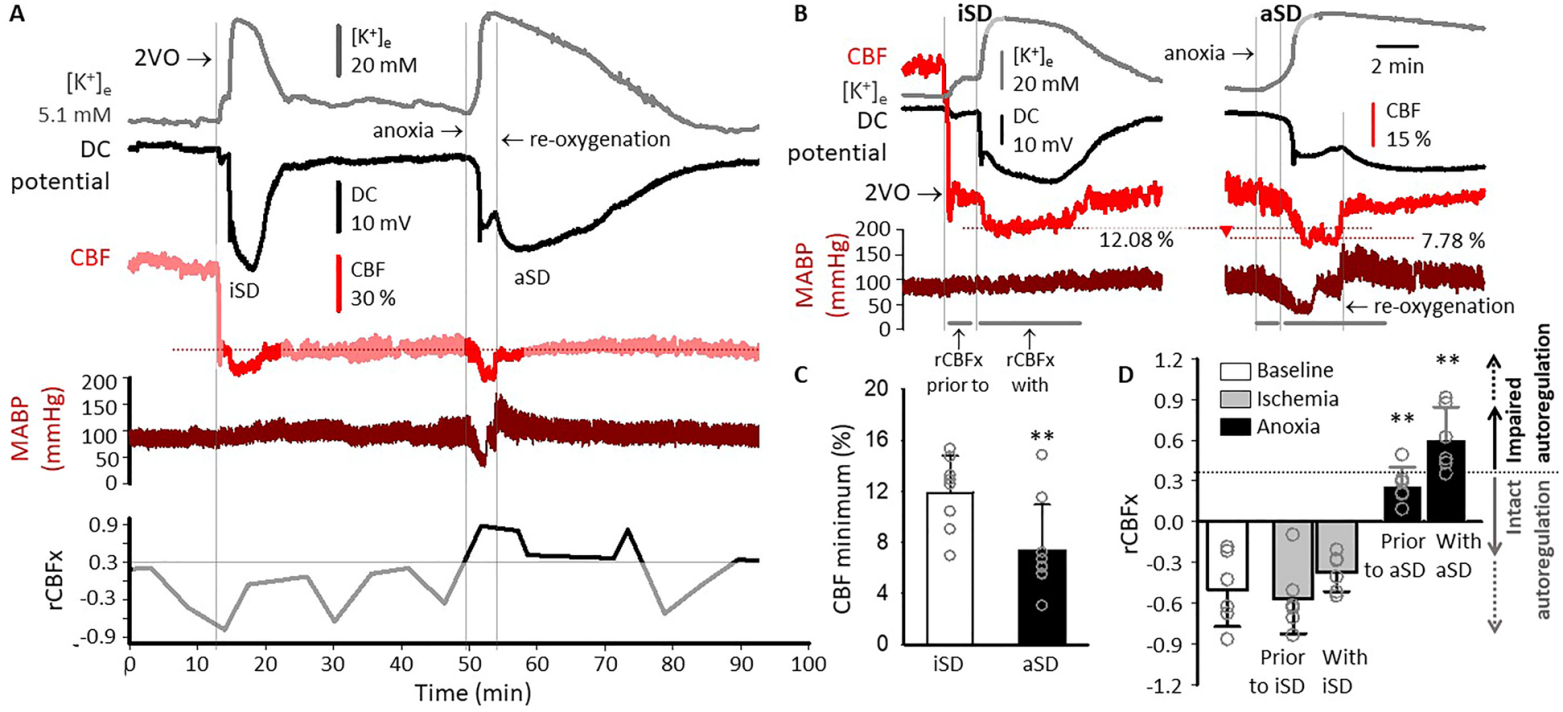
Impaired cerebrovascular autoregulation during anoxic spreading depolarization (aSD), in contrast with intact autoregulation with ischemic spreading depolarization (iSD). **a** Traces of a representative experiment demonstrate the DC potential signature of iSD in response to the permanent, bilateral occlusion of the common carotid arteries (2VO) and aSD under the episode of anoxia. Anoxia was maintained for 4 min during this experiment. Delayed repolarization after aSD evolved after reoxygenation. The cerebral blood flow (CBF) recording (red trace) shows transient hypoperfusion with iSD and aSD. The hypoperfusion transients are highlighted (brighter color) in such a way that level CBF segments before and after the hypoperfusion transients are included for reference. The highlighted periods are of equal duration. Mean arterial blood pressure (MABP) (deep red trace) gradually elevated after 2VO onset but dropped shortly after anoxia induction. CBF reduction was time-locked with decreasing MABP after anoxia induction. The cerebrovascular autoregulatory index (rCBFx) (bottom trace; Hinzman et al. [21]) indicated dysfunctional cerebrovascular autoregulation (i.e., rCBFx > 0.03) in association with aSD. **b** The iSD and aSD in the representative recording are shown at a higher resolution to appreciate a lower CBF minimum with aSD compared with iSD (red arrowhead) and to demonstrate the synchronous changes in CBF and MABP with aSD. The periods serving as the basis for rCBFx calculations are indicated with horizontal gray lines below the MABP trace. **c** The CBF minimum over the event-related hypoperfusion transient was deeper with aSD than with iSD. **d** Quantitative evaluation of rCBFx calculated for a period of 60 s during baseline (i.e., before ischemia induction, 2VO) prior to iSD (after ischemia induction but before iSD) or prior to aSD (after anoxia initiation but before aSD) and with iSD or aSD (during the DC deflection). Note that autoregulation was weakening after anoxia initiation and proved to be fully impaired during aSD. In contrast, autoregulation was intact under iSD. Data in **b–c** are given as mean ± stdev. Statistical analysis relied on a one-way analysis of variance model run in SPSS; *p* < 0.01**. In **d** a Bonferoni post hoc test was applied. *p* < 0.01** vs. baseline and ischemia. 2VO, two-vessel occlusion, DC direct current, stdev, standard deviation

Laser Doppler flowmetry in combination with electrophysiology showed that iSD and aSD were associated with a brief hypoperfusion (Fig. 2a). The CBF minimum reached during the event-associated hypoperfusion transient was significantly lower with aSD than with iSD (7.4 ± 3.6 vs. 11.9 ± 2.9%, respectively) (Fig. 2b), although they were of similar duration (202 ± 113 and 189 ± 80 s, respectively). Detailed analysis of CBF changes in relation to MABP revealed meaningful differences between the flow variations with iSD and aSD. In particular, the reduction of CBF was independent of MABP with iSD, whereas the drop of CBF tightly followed that of MABP reduction with aSD (Fig. 2c). Cerebral autoregulation, which was functional before and under ischemia, was weakening after anoxia initiation prior to aSD. This was reflected by rCBFx (0.27 ± 0.14) approaching the threshold of autoregulatory failure (0.3) (Fig. 2c). Autoregulatory failure became obvious shortly before and under aSD, which was substantiated by rCBFx (i.e., 0.61 ± 0.24 during aSD) exceeding 0.3, taken as the upper limit of intact autoregulation [21]. With iSD, CBF dropped in the face of increasing MABP, yielding negative rCBFx values (− 0.38 ± 0.14 during iSD).

**Fig. 3:**
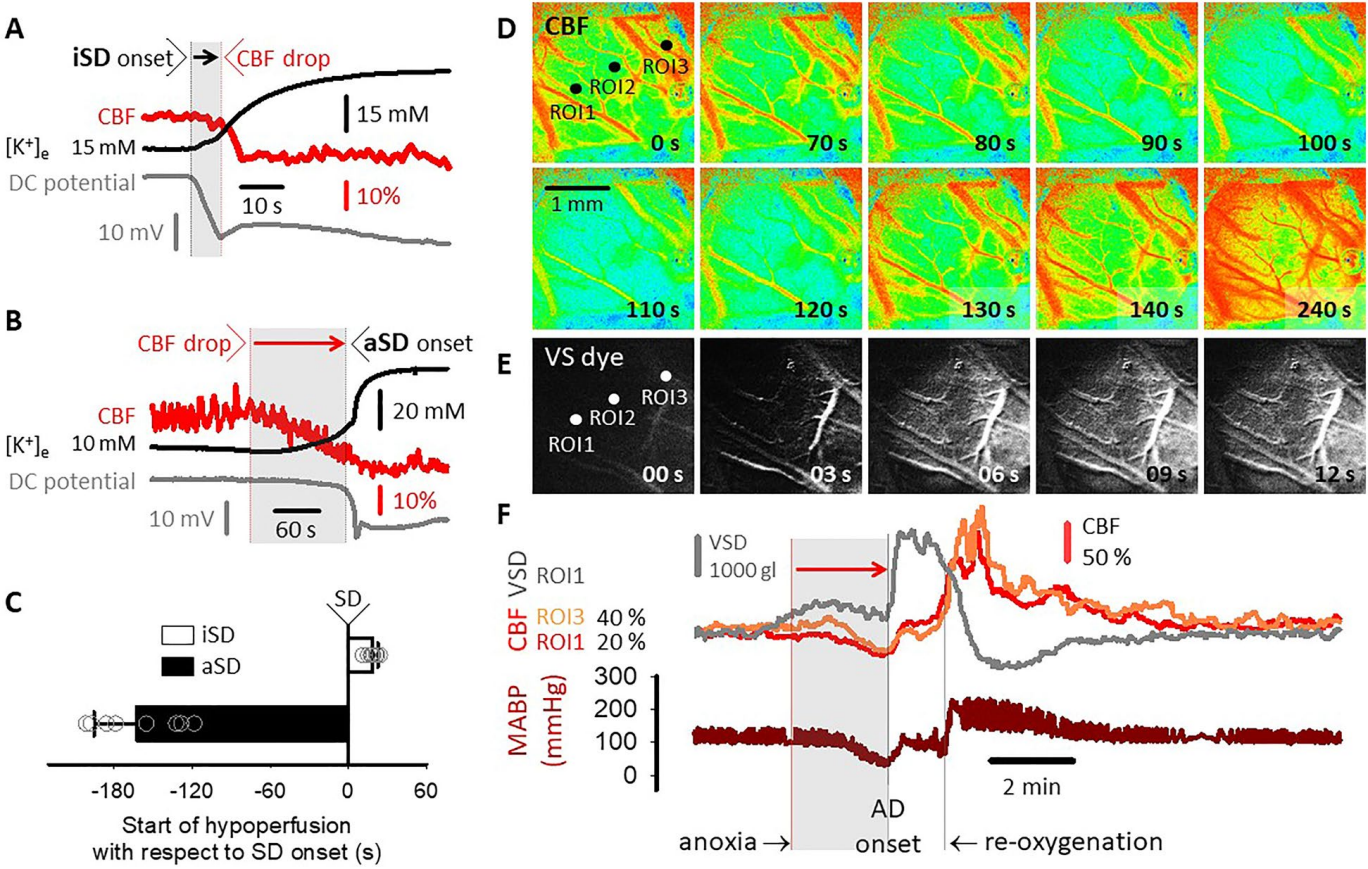
The temporal sequence and the latency between spreading depolarization (SD) and the associated transient hypoperfusion. **a** Representative traces (cerebral blood flow [CBF], extracellular K^+^ concentration [K^+^]_e_, and direct current [DC] potential) display the delay of the local drop of CBF with respect to ischemic SD (iSD) at a high resolution. The gray-shaded box and black arrow highlight the latency. **b** In the same representative experiment, the start of the more gradual reduction of CBF preceded anoxic SD (aSD) onset by minutes. The gray-shaded box and red arrow highlight the delay of aSD. **c** The start of the transient hypoperfusion with respect to iSD or aSD onset. Data are shown as mean ± stdev. **d** CBF maps calculated with LASCA represent CBF changes associated with an aSD event. Three regions of interest (ROI) were placed on the image sequences along the propagation of an iSD prior to aSD. **e** Voltage-sensitive dye (VS-dye) images demonstrate the evolution of aSD shortly after anoxia initiation. Note the different time scale with respect to Panel D, due to the difference in temporal resolution of the events. **f** Variations of CBF at ROI1 (red) and ROI3 (orange) under the episode of anoxia were synchronous and in tight relation to changes of mean arterial blood pressure (MABP). Note that hypoperfusion evolved in response to anoxia initiation and preceded aSD onset (VS dye trace in gray). CBF elevation after reoxygenation minutely followed the increase of MABP. LASCA, laser speckle contrast analysis

At a high resolution, the sequence of iSD and aSD and the associated flow reduction proved to be opposing. While the CBF drop occurred at a short delay after iSD (18 ± 4 s), CBF reduction preceded aSD by minutes (− 162 ± 33 s) (Fig. 3a–c). A series of CBF maps obtained with LASCA (Fig. 3d) and the spatio temporally matching VS-dye image sequence (Fig. 3e), confirmed that the hypoperfusion transient with aSD preceded the depolarization, rather than evolving as a consequence of aSD, and was simultaneous in the field of view (Fig. 3f). Any subsequent flow increase appeared to be also passive and follow the elevation of MABP minutely.

## Discussion

The conversion of the ischemic penumbra to the infarction is a fundamental process in the progression of ischemic brain injury, which worsens clinical outcome. Accumulating experimental and clinical evidence suggest that the irreversible damage in the penumbra is mediated by recurring SDs [18], and impaired cerebrovascular autoregulation contributes to the progression of secondary injury [1]. In the present ischemia model, we aimed to recapitulate the evolution of SDs and the associated CBF response in penumbra tissue and monitored the efficacy of cerebral autoregulation simultaneously. We relied on a rat global forebrain ischemia model to create a perfusion deficit, which is uniform over the entire cortex and comparable to that in the ischemic penumbra [24].

The experiments were conducted under isoflurane anesthesia, which may be perceived as a limitation of the study. Volatile anesthetics, including isoflurane used here, are widely known to dilate cerebral vessels, and may impair cerebral autoregulation [28]. A previous experimental study, for instance, found a dose-dependent effect of isoflurane administration on cerebral autoregulation. Cerebral autoregulation was preserved under 1.5% isoflurane, but CBF followed MABP passively at an isoflurane concentration of 2.8% [29]. Here, isoflurane concentration was maintained around 1.2% during data acquisition, and the calculated rCBFx indicated no cerebral autoregulatory dysfunction during baseline and the event-free period of ischemia prior to anoxia (Fig. 2a, d).

In our model, SD occurred in response to a sharp drop of CBF due to vascular occlusion (iSD), and to a further transient anoxic episode (aSD), intended to cause a sudden worsening of supply–demand mismatch, a known trigger of SD [8]. iSD and aSD were of the same amplitude but displayed some clear dissimilarities. iSD typically set off from a supraphysiological, level baseline [K^+^]_e_ (9–15 mM). In contrast, aSD was superimposed on a gradual rise of pathological [K^+^]_e_ (18–23 mM), seen in previous studies during N_2_ inhalation or after cardiac arrest [30, 31]. The common condition to favor the slow rise of [K^+^]_e_ leading to SD in our experiments and in those presented in previous reports is, apparently, anoxia. As anoxia impairs ATP-dependent ion pumps, the mechanism behind the slow [K^+^]_e_ elevation was suggested to be the failing function of the Na^+^/K^+^ ATPase [30, 31]. Also, the gradual [K^+^]_e_ shift may be the reason for the longer latency to aSD, compared with iSD, as seen here.

aSD evolved here in response to an episode of anoxia superimposed on ischemia, at uninterrupted cardiac function. The cortical tissue repolarized after restoring oxygen supply, inferring that at least some of the tissue underwent reversible crisis and did not reach the so called commitment point (the initiation of irrevocable neuronal injury under persistent depolarization) [32, 33]. Indeed, patch clamp recordings in live brain slice preparations exposed to transient oxygen–glucose deprivation also prove that a considerable proportion of cortical neurons survive oxygen–glucose deprivation sustained as long as 10 min if the physiological condition is subsequently restored [34]. In addition, randomly distributed pyramidal neurons in the core region were found to survive focal ischemic stroke in the mouse, as evidenced by their intact electrophysiology and dendritic morphology 12 h after ischemia onset [35]. This may be thought provoking and support the concept recently put forward that the onset of terminal depolarization may not necessarily indicate inevitable, commencing brain death in patients [36, 37].

SD has been understood to compromise tissue viability by the insufficiency of the associated CBF response, particularly when SD is coupled with spreading ischemia [16, 17, 38]. In the present study, we identified hypoperfusion transients with SD, accepted as the signature of spreading ischemia (Fig. 2). Spreading ischemia in response to SD is thought to be brought about by active vasoregulation. Cerebrovascular smooth muscle cells constrict in response to the massive accumulation of K^+^ more than 20 mM in the extravascular or perivascular space with SD, aggravated by the restricted availability of nitric oxide [16, 19, 20]. Further, clinical studies have demonstrated that spreading ischemia may coincide with the dysfunction of cerebrovascular autoregulation after traumatic brain injury or subarachnoid hemorrhage [21, 22]. Also, an experimental study linked SD-related spreading ischemia to autoregulatory failure in the context of hypovolemic hypotension [38]. These reports speculated that the cerebrovascular autoregulatory deficit may predispose the tissue to spreading ischemia in response to SD [22, 38], or that SD itself may be the underlying cause of both the CBF reduction and autoregulatory failure with SD [21].

Here, we made the observations that the hypoperfusion transient with iSD occurred at functional cerebrovascular autoregulation (i.e., MABP remained in the range of autoregulation, around 90–110 mm Hg, and variations in CBF and MABP were unrelated). In contrast, aSD was coincident with autoregulatory failure standing for decreasing CBF along falling MABP. This is consistent with the observation that a rapid decrease of systemic blood pressure disables the autoregulatory response [39]. Of note, hypoxia as measured here (arterial pO_2_ down to 35.4 ± 12.9 mm Hg) is a potent dilator in the cerebral circulation when CBF regulation is intact [40]. CBF elevation linked to the anoxic episode was not seen here, which substantiates the impairment of cerebral vasoregulation further. MABP was decreasing apparently because the systemic anoxia compromised cardiac function. When autoregulation is intact, the cerebral microvasculature is expected to compensate for the MABP reduction by reducing cerebrovascular resistance through arteriolar vasodilation. A previous study of ours demonstrated that cerebral arteriolar vasodilation in compensation for ischemic hypoperfusion was functionally impaired in old as compared with young rats [41], possibly because of the age-related excessive production of reactive oxygen species [42]. Taken together, the loss of autoregulation on anoxia here may be explained by the rapid drop of MABP and the lack of effective CBF compensation due to the impairment of cerebral arteriolar dilation.

The reduction of CBF concomitant with the arising hypotension exacerbated ongoing ischemia, also worsened by anoxia. Importantly, both the autoregulatory failure and the CBF reduction started to develop with anoxia onset, minutes before aSD occurrence (Figs. 2c, 3). Finally, the hypoperfusion transient preceding aSD was not spreading but evolved uniformly over the field of view in our imaging experiment (Fig. 3c). On the basis of our present results, we posit that hypoperfusion linked to aSD evolving in the face of dysfunctional autoregulation (1) must be consequential to the loss of autoregulation, (2) is passive in flow reduction, rather than representing actively regulated vasoconstriction as with spreading ischemia, and (3) actually may contribute to the generation of aSD [8], rather than being initiated by the depolarization. This notion may introduce an important point of consideration for the interpretation of the SD-coupled CBF reduction when cerebrovascular autoregulation is dysfunctional. Finally, the lack of any type of CBF response with SD during an impairment of cerebral autoregulation may be perceived as an indicator of a serious injury of neurovascular coupling.

## Conclusions

Taken together, we suggest that transient CBF reduction in relation to SD events may have two variants: (1) spreading ischemia under preserved autoregulation [16], which propagates across the cerebral cortex coupled to SD and reflects active vasoconstriction related to the high concentration of extracellular K^+^ [20], and (2) a short flow reduction linked to failing autoregulation, which passively follows a drop of blood pressure, involves a large cortical tissue volume, and predisposes the cortex to an SD event. We propose that the dysfunction of cerebrovascular autoregulation simultaneous with hypotension transients poses a substantial risk for SD occurrence and may cause the complete loss of neurovascular coupling. The causality outlined here is expected to generate further investigation into the link between cerebrovascular autoregulatory failure and SD generation in the acutely injured human brain.

## Data Availability

The data sets generated or analyzed during the current study are available from the corresponding author on reasonable request.
